# Rural governance capacity and land-use efficiency: A comparative analysis of rural modernization in China and India

**DOI:** 10.1371/journal.pone.0356874

**Published:** 2026-08-25

**Authors:** Yangyi Qu, Jie Tang, Hailong Wang

**Affiliations:** 1 School of Sociology and Ethnology, University of Chinese Academy of Social Sciences, Beijing, China; 2 School of Marxism, Chongqing University, Chongqing, China; Macau University of Science and Technology, MACAO

## Abstract

This study examines how village-level governance capacity shapes rural modernization outcomes across China and India from the perspective of Common-Pool Resource (CPR) theory and the Institutional Analysis and Development (IAD) framework. A multidimensional evaluation system comprising 32 indicators across four dimensions—Infrastructure and Public Services, Governance and Participation, Economic Development, and Habitation and Ecology—was constructed for 60 villages in the two countries. The entropy weight method and principal component analysis (PCA) were employed to develop a composite core capacity factor, while cross-sectional OLS regression models were used to examine institutional moderation and mediation mechanisms. The results show that village core capacity significantly improves rural living satisfaction, indicating that effective governance coordination and institutional capacity are critical for enhancing land-use efficiency. Infrastructure and Public Services further function as an important mediating mechanism through which governance capacity is translated into welfare outcomes. The findings also show that national institutional context moderates the relationship between core capacity and rural modernization outcomes. China’s centralized governance model achieves stronger infrastructure coordination and public service delivery, whereas India’s decentralized governance structure exhibits greater variation in implementation effectiveness across villages. In both countries, institutional capacity to reduce transaction costs and coordinate collective action is more important than economic scale alone for sustainable rural modernization. In addition, urban-rural fringe villages display significantly higher modernization performance than purely agricultural villages. This research enriches the cross-national comparative study of rural transformation, verifies the explanatory power of CPR theory in internationally comparative macro institutional contexts, and provides a structured evaluation framework for rural modernization in emerging economies.

## Introduction

India and China, home to more than one-third of the world’s population, have undergone profound economic and institutional transformations over the past four decades. As two of the largest developing economies, their rural modernization trajectories have drawn sustained scholarly attention [1]. Comparative research has examined differences in economic growth [2], digital development [3], industrialization patterns [4], healthcare systems [5,6], foreign direct investment [7], and technological advancement [8]. Yet despite this extensive body of literature, systematic cross-national analyses of rural modernization at the village level remain relatively limited. Existing studies often focus on macroeconomic performance or sector-specific outcomes, leaving insufficient understanding of how governance arrangements, economic resources, and land-use practices interact within rural communities.

Rural modernization in both countries is not merely a process of income growth but a multidimensional restructuring of governance systems, collective participation, public service provision, land allocation, and ecological management. China has advanced rural revitalization largely through centralized coordination and state-led infrastructure provision, whereas India has institutionalized decentralized governance through constitutionally empowered Panchayats. These contrasting institutional arrangements offer a valuable comparative setting for examining a broader theoretical question: how do governance structures and economic development interact to shape land-use efficiency and rural modernization outcomes?

Much of the existing literature relies on descriptive comparisons or single-country case studies that rank development performance without identifying the mechanisms underlying observed differences. Modernization is frequently proxied by economic indicators alone, overlooking ecological sustainability, habitation conditions, and collective governance capacity. As a result, it remains unclear whether cross-national disparities reflect differences in economic scale, institutional design, or the interaction between governance capacity and resource endowments. Addressing this gap requires both multidimensional measurement and empirical testing of institutional-economic linkages.

The study is guided by two central research questions: first, what structural differences characterize village modernization patterns in China and India across multiple functional dimensions; and second, how do governance arrangements and economic development interact to explain variation in land-use efficiency within and across the two countries? By situating the analysis within broader debates on rural transformation and common-pool resource governance, this research contributes to understanding how institutional coordination shapes development outcomes in emerging economies, and serves as an applied extension of CPR theory to cross-national comparisons. Rather than asking which governance model is inherently superior, the study examines how institutional contexts condition the conversion of economic resources into coordinated and sustainable land-use outcomes.

The remainder of the paper proceeds as follows. The Literature review section synthesizes existing scholarship related to rural modernization, governance capacity, and land-use efficiency, develops our theoretical framework grounded in CPR perspectives, and formulates testable research hypotheses. The Data and Methodology section describes our sampling strategy, survey data, indicator construction, entropy-weighting workflow, and regression model specifications. The Results section reports the descriptive entropy-based evaluation outputs alongside baseline regression, moderation, mediation, and robustness estimation results. The Discussion section interprets the empirical findings and unpacks their theoretical and practical significance. Finally, the Conclusion section summarizes core findings, derives policy implications, acknowledges study limitations, and suggests directions for future research.

## Literature review

### Ostrom’s CPR theory and its evolution

Elinor Ostrom’s pioneering research on Common-Pool Resources (CPR) contested the inevitability of the “tragedy of the commons,” demonstrating that local, self-governing institutions could achieve sustainable management of shared resources through collective action [9]. Her eight design principles—emphasizing clearly defined boundaries, proportional costs and benefits, collective choice arrangements, monitoring, and conflict resolution—offer a rigorous framework for analyzing micro-level resource governance [10]. The CPR theory posits that the efficacy of resource planning, such as land allocation, is fundamentally enhanced when internal governance rules are designed by the users themselves, as endogenous rules foster shared norms and reduce monitoring and enforcement costs. This perspective suggests that robust local participation acts as a catalyst for land-use efficiency by ensuring planning aligns with local needs and mitigating free-rider problems. The CPR theory has been applied in diverse contexts, ranging from fisheries and forests [11] to groundwater management [12], highlighting its relevance to rural socio-ecological systems.

Beyond the initial principles, Ostrom’s later work on Institutional Diversity and the Institutional Analysis and Development (IAD) framework emphasized that “nested enterprises” must function across multiple scales [13]. This evolution acknowledges that while local rules are vital, the macro-institutional environment significantly shapes the effectiveness of local collective action. For instance, the success of CPR management depends on the alignment of technological, economic, and institutional factors, where economic development can lower the information and negotiation costs associated with collective action. Importantly, Ostrom’s later work increasingly emphasized that the ultimate effectiveness of commons governance should be evaluated not only by resource preservation itself, but also by the extent to which institutional arrangements improve the welfare and long-term livelihoods of resource users [14]. In rural land systems, governance effectiveness is therefore reflected not merely in physical infrastructure provision, but in whether land allocation and collective resource management enhance villagers’ overall living conditions and satisfaction [15,16].

However, critiques have pointed out limitations in its scalability. Yoder et al. argue that the CPR theory’s diagnostic focus on local collective action struggles to address large-scale dilemmas, such as climate change [17]. Choe and Yun further contend that Ostrom’s framework treats resource “excludability” and “rivalry” as static physical attributes, overlooking their dynamic social construction under different historical and institutional contexts [18]. Although recent research has explored extensions to urban land [19] and regional governance [20], a significant gap remains in applying CPR principles to macro-scale comparative analyses of national rural governance models, especially between major economies like China and India.

### Rural governance capacity, land-use efficiency, and welfare outcomes

Infrastructure and public services are the cornerstones of rural modernization, influencing land-use efficiency through their interrelationships with governance, economic structures, and ecological systems.

China’s rural infrastructure development is predominantly state-driven, leveraging fiscal centralization and land finance [21]. Strategic investments in transportation [22], electrification [23], and irrigation [24] have enhanced agricultural productivity and non-farm incomes. However, the quality of governance significantly determines local outcomes. Liu et al. reveal that village governance, more than project design, determines the quality of infrastructure [25], while Qin et al. identify transportation access and grassroots partnerships as drivers of village economic diversification [26]. Despite progress, imbalances persist: Shi et al. note the rapid development of infrastructure and public services alongside lagging governance and ecological modernization, resulting in spatial inequalities [27].

India’s rural infrastructure development reflects decentralized fiscal structures and spatial disparities. While large-scale programs such as the Pradhan Mantri Gram Sadak Yojana (PMGSY, rural roads) enhance agricultural GDP and non-farm employment [28], progress is uneven. Weak local governance restricts the development of third-tier (village-level) institutions essential for CPR management [29]. Studies also highlight quality deficiencies: unreliable electricity access undermines non-agricultural incomes [30], and underfunded maintenance limits the benefits of rural connectivity [31].

Crucially, the CPR theory emphasizes that collective mobilization, which is stronger in China, explains the divergent infrastructure outcomes: Gürel attributes China’s superior performance to its historical ability to mobilize rural labor and finance for public goods, a capacity hampered in India by entrenched local elites [32], which may also involve systemic discrimination against tribal and low-caste groups. These cross-national differences in governance coordination, infrastructure provision, and public service delivery suggest that land-use efficiency is ultimately shaped by broader institutional capacity rather than infrastructure inputs alone.

Land-use efficiency (output per unit area) in rural areas is increasingly understood as a multidimensional governance outcome rather than a purely physical infrastructure outcome. Existing studies suggest that efficient land use depends on whether governance systems can effectively coordinate infrastructure provision, public services, ecological protection, and economic opportunities within limited spatial resources [33–37]. From the perspective of CPR theory, land governance efficiency ultimately lies in improving the welfare of resource users through effective institutional coordination and collective action [9,13,14]. Recent rural development studies increasingly employ subjective well-being and resident satisfaction as comprehensive outcome indicators reflecting the effectiveness of governance, infrastructure provision, and public resource allocation [38–41]. Accordingly, villagers’ living satisfaction reflects the extent to which land allocation, spatial planning, and public resource management successfully meet local livelihood needs. Infrastructure and public services, while also representing one observable dimension of rural modernization, therefore constitute important intermediary mechanisms through which governance capacity influences rural welfare and land-use efficiency, rather than efficiency outcomes themselves [42–44].

### Core capacity and multidimensional rural modernization

Recent governance research increasingly conceptualizes governance capacity as a measurable institutional performance system involving participation, accountability, coordination, and public service delivery [45–48]. Rather than treating governance as a purely political abstraction, contemporary governance studies emphasize indicator-based assessment frameworks capable of evaluating institutional effectiveness across multiple dimensions [49–51]. Within rural development research, governance quality has been closely associated with infrastructure provision, collective participation, and local public goods performance [25,52].

Rural modernization is similarly understood as a multidimensional transition rather than a purely economic process [53]. Existing studies have therefore proposed composite evaluation systems integrating governance effectiveness, economic development, infrastructure conditions, ecological sustainability, and social well-being [27,54–57]. Such multidimensional approaches are particularly important in comparative studies of emerging economies, where institutional arrangements shape the coordination of land use, public service provision, and collective action outcomes.

Building upon Ostrom’s CPR framework and the IAD approach [9,13], this study conceptualizes rural modernization as a multidimensional institutional transformation shaped by an integrated village-level core capacity [53]. This core capacity is jointly constituted by Governance and Participation, Economic Development, and Habitation and Ecology, which together determine the ability of rural governance systems to coordinate land allocation, infrastructure provision, ecological management, and public service delivery [9–11,13,25,33,58,59]. By integrating these dimensions into a unified analytical framework, this study operationalizes CPR theory within a macro-comparative rural modernization context and links institutional governance capacity to measurable modernization outcomes [45,46,49,54,55].

Helpful in this context, entropy-based weighting methods have been widely applied in sustainability assessment, ecological evaluation, and institutional performance measurement because they reduce subjective bias in indicator weighting and capture informational heterogeneity across variables [60–64]. Compared with expert-based weighting approaches, the entropy method provides a more objective governance-assessment framework for evaluating multidimensional rural modernization processes. In this study, entropy weighting is adopted to operationalize institutional performance across the four dimensions of Infrastructure and Public Services, Governance and Participation, Economic Development, and Habitation and Ecology. To further reduce potential multicollinearity among highly correlated modernization dimensions, the study subsequently employs principal component analysis (PCA) to synthesize Governance and Participation, Economic Development, and Habitation and Ecology into a unified core capacity factor for regression analysis.

### Critical research gap

Despite substantial research on rural modernization and common-pool resource governance, several important gaps remain.

First, although Ostrom’s CPR theory and the IAD framework have been widely applied to micro-level collective action and local resource management, limited research has operationalized these theories within comparative analyses of rural governance systems across different national institutional contexts [9,13]. Existing studies largely emphasize localized commons management while paying insufficient attention to how broader institutional environments shape the conversion of governance capacity into rural development outcomes across different national institutional contexts.

Second, comparative studies of China and India have predominantly focused on macroeconomic growth, decentralization, or sectoral policy performance [1–8,29], while relatively few studies systematically examine how public service provision, governance arrangements, economic development, and ecological conditions interact at the village level [32]. In particular, the relationship between governance participation and land-use efficiency remains insufficiently theorized and largely empirically untested in cross-national rural modernization research.

Third, existing rural modernization studies often rely on either descriptive institutional analysis or single-dimensional economic indicators [27,54–57,65]. Although multidimensional evaluation systems have gradually emerged, limited research integrates governance measurement frameworks with land-use efficiency assessment within a unified analytical model. Moreover, few studies operationalize governance and modernization through entropy-based multidimensional indicators capable of capturing cross-village institutional variation in comparative settings [60–64].

To address these gaps, this study develops a multidimensional comparative framework linking Infrastructure and Public Services, Governance and Participation, Economic Development, and Habitation and Ecology within an entropy-based institutional assessment model. Building upon CPR theory and the IAD framework, the study further conceptualizes village core capacity as an integrated institutional foundation shaping rural modernization outcomes. Rather than treating land-use efficiency as a purely physical infrastructure outcome, this study conceptualizes land-use efficiency from a welfare-oriented perspective. Accordingly, rural living satisfaction is employed as a proxy reflecting the effectiveness of governance coordination, public service provision, and collective resource allocation. Infrastructure and Public Services are further examined as an intermediary mechanism through which village core capacity enhances welfare-oriented land-use efficiency. By empirically examining these relationships across villages in China and India, this research extends CPR theory from localized commons management toward macro-comparative institutional analysis. In doing so, the study contributes to the growing literature on governance capacity, rural modernization, and land-use transition pathways in emerging economies.

### Study hypotheses

Following CPR theory, rural land-use efficiency is understood not merely as the physical expansion of infrastructure, but as the extent to which land governance arrangements improve the welfare outcomes of resource users through effective coordination of public resources and collective action. Accordingly, this study employs rural living satisfaction as a proxy for land-use efficiency, reflecting villagers’ overall evaluation of governance effectiveness, infrastructure provision, public services, and living conditions. Within this framework, Governance and Participation, Economic Development, and Habitation and Ecology are integrated into a unified concept of village core capacity, which captures the institutional and developmental foundations of rural modernization. Based on this theoretical perspective, the following hypotheses examine how core capacity, national institutional context, and spatial characteristics shape land-use efficiency outcomes across China and India.

#### Collective action and governance efficacy.

Ostrom’s CPR theory emphasizes that effective collective action depends on institutional arrangements that reduce monitoring, coordination, and enforcement costs through locally embedded governance mechanisms [9,10]. Within the IAD framework, participatory governance and collective-choice arrangements improve the capacity of local actors to coordinate shared resource management and public goods provision [13]. Governance effectiveness literature similarly suggests that institutional quality, accountability, and participation are closely associated with public service delivery performance, collective welfare, and rural development outcomes [45,52,66–69].

Recent rural development studies further argue that the effectiveness of land governance should ultimately be evaluated not only by physical infrastructure expansion, but also by the extent to which governance arrangements improve the well-being and satisfaction of rural residents through better public services, ecological conditions, and livelihood opportunities [38–41]. Subjective welfare indicators such as rural living satisfaction are therefore increasingly employed as comprehensive outcome measures reflecting governance effectiveness and public resource allocation efficiency in rural modernization processes [44,70].

Building on these theoretical and empirical insights, this study argues that villages with stronger core capacity—integrating Governance and Participation, Economic Development, and Habitation and Ecology—are better able to coordinate collective action, allocate public resources, and improve rural welfare outcomes.

**Hypothesis 1 (H1):** Core capacity positively improves land-use efficiency proxied by rural living satisfaction.

#### Macro-institutional environments as moderators.

Although local governance mechanisms are important, the IAD framework emphasizes that institutional outcomes are embedded within broader multi-level governance systems [13]. National institutional arrangements shape how local governance capacity is translated into land-use coordination and infrastructure provision. Existing studies suggest that state-centered institutional coordination may reduce implementation fragmentation and lower transaction costs in large-scale development programs [71,72]. In China, public land ownership and centralized administrative coordination may facilitate more integrated infrastructure planning and collective resource mobilization [71,73]. Comparative studies of decentralization further note that institutional effectiveness depends not only on formal decentralization itself, but also on administrative coordination capacity, fiscal support, and local accountability mechanisms [29,74–76]. Decentralized governance systems may also improve responsiveness to local needs and strengthen participatory accountability under favorable institutional conditions [77,78]. By contrast, fragmented property-right structures and decentralized administrative arrangements may increase coordination costs and institutional fragmentation in land governance systems [79]. Research on India’s rural governance has shown that decentralized institutions alone do not necessarily guarantee effective implementation capacity, particularly under uneven fiscal and administrative conditions [29,74–76].

Rather than assuming the superiority of a specific governance model, this study proposes that national institutional contexts condition the extent to which village core capacity can be translated into land-use efficiency outcomes. Therefore, this study proposes:

**Hypothesis 2 (H2):** National institutional context moderates the relationship between core capacity and land-use efficiency.

#### Spatial characteristics and resource systems.

Ostrom’s CPR theory further suggests that governance effectiveness is conditioned by the spatial characteristics of resource systems [9]. In rural modernization processes, urban–rural fringe zones represent transitional spatial environments where land scarcity, infrastructure demand, and economic diversification intensify pressures for coordinated land-use optimization [80,81]. Previous studies indicate that peri-urban regions often experience stronger infrastructure integration, higher land values, and more diversified economic activities compared with purely agricultural regions [80,82]. Land-use transition studies further show that spatial competition and infrastructure concentration in fringe zones often accelerate land-use optimization processes [83]. Under conditions of limited per capita arable land, governance systems may therefore face stronger incentives to optimize infrastructure allocation and improve land-use efficiency through spatial coordination.

Accordingly, urban–rural fringe villages are expected to exhibit higher levels of land-use efficiency than purely agricultural villages.

**Hypothesis 3 (H3):** Urban–rural fringe villages exhibit significantly higher land-use efficiency than agricultural villages, particularly under conditions of lower per capita arable land availability.

Beyond the three core hypotheses, this study further investigates the internal mechanism linking village core capacity and land-use efficiency. Specifically, Infrastructure and Public Services is examined as a potential mediating channel through which core capacity enhances rural living satisfaction, thereby revealing how village core capacity is translated into welfare-oriented land-use efficiency outcomes through Infrastructure and Public Service.

## Data and methodology

### Data collection

The data for this study were acquired from 60 villages in China and India, selected based on a systematic, purposive sampling framework. The sample size of 60 villages is methodologically justified by the principles of theory-driven comparative research [52,84]. Unlike large-N correlational studies that prioritize statistical power for population inference, hypothesis-testing research in cross-national rural studies emphasizes theoretical representativeness—the degree to which cases exemplify the core institutional variants under investigation [85]. This approach is particularly appropriate given the prohibitive cost, logistical complexity, and data access challenges associated with collecting primary, objective village-level data across two geographically vast and institutionally diverse developing economies [72].

Provinces/states were categorized into three tiers (upper, middle, and lower) according to their official per capita income rankings in 2021 from their respective official data to represent distinct income levels, as detailed in Table 1, ensuring a diversified socioeconomic representation. While acknowledging potential COVID-19 impacts on absolute GDP values, three methodological safeguards ensure sampling validity. First, relative intra-country economic rankings remained highly stable despite pandemic disruptions. Second, sampling prioritized these stable ordinal tiers—not absolute GDP values—to capture enduring socioeconomic gradients. Third, village-level modernization indicators reflect long-term investments less susceptible to short-term GDP volatility than national aggregates. Urbanized regions such as Beijing, Shanghai, Zhejiang, and Guangzhou Province in China were excluded to focus on traditional rural villages, which lag relatively and need modernization utmost.

**Table 1:** List of stratified sampling provinces for the questionnaire survey.

Country	Province/State	Per capita (2021)		Domestic GDP ranking
		RMB (¥)	Rupee (₹)	
China	Chongqing	87000	996,898	8/31
	Shanxi	64700	741,371	17/31
	Gansu	40900	468,657	31/31
India	Tamil Nadu	18322	209,944	9/32
	Punjab	12944	148,320	17/32
	Bihar	3766	43,153	32/32

Notes:

1. Rankings are out of 31 provincial-level divisions in China (including provinces, autonomous regions, and direct-controlled municipalities) and 32 administrative units in India. Sources: National Bureau of Statistics of China; Ministry of Statistics and Programme Implementation of India.

2. The average exchange rate of RMB to rupee was 1:11.4586 in 2021. Conversion results are rounded to the nearest whole number.

The final sample includes one province/state from each tier in both countries. Ten villages of each province/state, with 30 villages total per country, the village sampling tries to ensure inclusion of 3 villages with good governance, 4 general, and 3 poor, according to their official, media, or social reputation. Two complementary instruments were implemented universally: a leader-administered survey documenting objective village characteristics and a household survey measuring service satisfaction. All 60 villages contributed both leader responses and 30 villager responses (questionnaires are available in S1 File). Verbal informed consent was obtained from all participants after explaining study objectives, data anonymity, and voluntary participation rights.

Rural living satisfaction in India was measured using a single validated survey item targeting village‑level governance satisfaction: “Generally, are you satisfied with the management of your village panchayat?” Responses were recorded on a five‑point Likert‑type scale: 1 = Very satisfied, 2 = Relatively satisfied, 3 = Neutral (Just so so), 4 = Relatively dissatisfied, and 5 = Very dissatisfied. For empirical analysis, individual villager responses were aggregated to the village level by calculating the mean satisfaction score per village, representing overall rural welfare outcomes and land‑use efficiency at the village level.

In total, 843 and 874 valid questionnaires were actually collected in China and India, respectively, supplemented by 30 completed leader questionnaires per country (see Tables 2 and 3). This variance stemmed from low household occupancy due to outmigration, language barriers inhibiting effective participation by elderly residents, and survey refusals. Questionnaire data for both countries are available in S2 and S3 Files. Ethical approval was obtained from relevant institutional authorities for surveys conducted in China and India. The IDs and basic information of the villages surveyed are presented in Table 4.

**Table 2:** Basic demographic statistics of villagers surveyed in China.

Province	Units surveyed	Household respondents							
	Villages (leader questionnaires completed)	Households (Villager questionnaires completed)	Male	Female	Age above 60	Education			
						Primary school and below	Junior middle school	High school	Higher education
Chongqing	10	256	55.50%	44.50%	51.20%	62.50%	25.70%	6.30%	5.50%
Shanxi	10	300	50.00%	50.00%	57.70%	54.70%	29.70%	12.30%	3.30%
Gansu	10	287	63.10%	36.90%	38.70%	54.00%	30.00%	10.80%	5.20%
Total	30	843	56.10%	43.90%	49.20%	47.90%	37.50%	10.00%	4.60%

Note: In China’s high-outmigration provinces (Chongqing/Shanxi), daytime surveys captured more female respondents due to significant male labor migration, whereas in Gansu (low-outmigration), cultural norms prioritized male household spokespersons during interviews regardless of female presence.

**Table 3:** Basic demographic statistics of villagers surveyed in India.

State	Units surveyed	Household respondents							
	Villages (leader questionnaires completed)	Households (Villager questionnaires completed)	Male	Female	Age above 60	Education			
						Primary school and below	Junior middle school	High school	Higher education
Tamil Nadu	10	300	49%	51%	0%	25.0%	6.0%	14.0%	55.0%
Punjab	10	285	54.4%	45.6%	5.7%	14.7%	19.6%	38.2%	27.4%
Bihar	10	289	70.9%	29.1%	17.6%	27.0%	22.5%	26.0%	24.6%
Total	30	874	58%	42%	3.6%	22.3%	15.9%	25.9%	35.9%

Note: In India, Bihar’s stronger patriarchal constraints and female seclusion norms contrasted with Tamil Nadu/Punjab’s more inclusive social structures, where female participation in external interactions was comparatively higher.

**Table 4:** The IDs and basic information of the villages surveyed.

Country	Province/State	ID	Village	Village type	Geographical feature
China	Chongqing	1	Minxin	Agricultural	Hills
		2	Bayi	Agricultural	Plain
		3	Yongjin	Urban-rural fringe zone	Hills
		4	Dajing	Agricultural	Hills
		5	Taishan	Agricultural	Hills
		6	Longxing	Agricultural	Hills
		7	Jiangling	Agricultural	Hills
		8	Tai’an	Agricultural	Hills
		9	Libai	Agricultural	Hills
		10	Biqiao	Agricultural	Hills
	Shanxi	11	Nanyijian	Agricultural	Hills
		12	Zhuangze	Agricultural	Hills
		13	Pengpotou	Agricultural	Hills
		14	Zhukeng	Agricultural	Hills
		15	Jiajiapo	Agricultural	Hills
		16	Shanpotou	Agricultural	Hills
		17	Zhao	Agricultural	Hills
		18	Erlang	Agricultural	Mountains
		19	Geta	Agricultural	Hills
		20	Shuimotou	Agricultural	Hills
	Gansu	21	Dashuilang	Agricultural	Plain
		22	Xijishui	Agricultural	Plain
		23	Jiuzhi	Agricultural	Plain
		24	Yuchuan	Agricultural	Beach area
		25	Xinmin	Urban-rural fringe zone	Beach area
		26	Dongliang	Agricultural	Mountains
		27	Yongan	Agricultural	Beach area
		28	Yongchuan	Agricultural	Plain
		29	Chenzhuang	Urban-rural fringe zone	Plain
		30	Xingquan	Agricultural	Hills
India	Tamil Nadu	31	Kumilankulam	Agricultural	Plain
		32	Paratyankulam	Agricultural	Reservoir area
		33	Mustakurichi	Agricultural	Plain
		34	Appanoor	Agricultural	Reservoir area
		35	Kottaimedu	Urban-rural fringe zone	Reservoir area
		36	Narasipuram	Agricultural	Hills
		37	Madampatti	Urban-rural fringe zone	Hills
		38	Thennamanallur	Agricultural	Reservoir area
		39	Odanthurai	Agricultural	Reservoir area
		40	Tas Nagar	Urban-rural fringe zone	Reservoir area
	Punjab	41	Bhangerian	Agricultural	Plain
		42	Ayali Khurd	Agricultural	Plain
		43	Tharike	Urban-rural fringe zone	Plain
		44	Thakarwal	Urban-rural fringe zone	Plain
		45	Bulara	Urban-rural fringe zone	Plain
		46	Sarinh	Urban-rural fringe zone	Plain
		47	Ranish Kalan	Agricultural	Plain
		48	Rauli	Agricultural	Plain
		49	Tatariewala	Agricultural	Plain
		50	Dosanjh	Agricultural	Plain
	Bihar	51	Bahiro	Agricultural	Plain
		52	Jamira	Agricultural	Plain
		53	Piparahiya	Agricultural	Plain
		54	Gothahula	Agricultural	Plain
		55	Dhanupara	Agricultural	Plain
		56	Balua	Agricultural	Plain
		57	Bhakura	Agricultural	Plain
		58	Chatarsainpur	Agricultural	Plain
		59	Dhauandhua	Agricultural	Plain
		60	Udawantnagar	Agricultural	Plain

### Methodology

#### Indicator development and operationalization.

This study constructs a hybrid indicator framework combining international governance benchmarks with country-specific rural modernization standards. Global governance references include the World Governance Indicators (WGI), Sustainable Governance Indicators (SGI), and the United Nations Sustainable Development Goals (SDGs) [45,46,49]. For China, the framework incorporates rural modernization evaluation systems proposed by the Chinese Academy of Social Sciences and the Development Research Center of the State Council [54,55]. For India, indicators are aligned with the institutional provisions of the 73rd Constitutional Amendment and the Ministry of Panchayati Raj [61–63].

Based on these sources, a systematic framework comprising 32 indicators across four dimensions—Infrastructure and Public Services, Governance and Participation, Economic Development, and Habitation and Ecology—was established (Table 5). The framework prioritizes land-related and village-level indicators to ensure cross-national comparability while maintaining local institutional specificity.

**Table 5:** Source mapping of the Sino-Indian comparative indicator system for rural village modernization.

Dimension	Indicator	International Source	Chinese Source	Indian Source
Infrastructure and Public Services	Main village road	WDI (Infrastructure)		Eleventh Schedule (Roads, bridges)
	Village roads	WDI (Infrastructure)		Eleventh Schedule (Communication means)
	Streetlights	SDG (Energy)	State Council Framework (Electricity)	Eleventh Schedule (Electricity)
	Broadband penetration rate	SDG (Infrastructure)	State Council Framework (Access to the Internet)	
	Tap water coverage rate	SDG (Clean water)	CASS System (Water supply)	Eleventh Schedule (Drinking water)
	Small-scale water conservancy facilities		CASS System (Irrigation)	Eleventh Schedule (Irrigation)
	Medical clinics/health stations	SDG (Health)	CASS System (Healthcare access)	Eleventh Schedule (Health centers)
	Coverage rate of licensed doctors per 1,000 rural residents	WDI (Health workers)	CASS System (licensed doctors per 1,000 rural residents)	
Governance and Participation	Sources of public project funding	SDG (Economic growth)	CASS System (Fiscal disclosure)	
	Village supervision methods	WGI (Accountability)	CASS System (Democracy)	
	Public meeting spaces	SGI (Democratic government)	CASS System (Democracy)	
	Villager voting rate	WGI (Voice & Accountability)	CASS System (Electoral participation)	
	Rate of villagers’ awareness of rights/information	SGI (Justice and strong institution)	CASS System (Democracy)	
	Rural amateur cultural organizations	SGI (Social sustainability)	CASS System (Cultural consumption)	Eleventh Schedule (Cultural activities)
	Major festival and celebration activities	SGI (Social sustainability)	CASS System (Cultural consumption)	Eleventh Schedule (Cultural activities)
	Fitness facilities	SDG (Well-being)	State Council Framework (Infrastructure)	
	Kindergartens/nurseries	SDG (Education)	State Council Framework (Education)	Eleventh Schedule (Education)
Economic Development	Annual income of village-run enterprises and collective economies	SDG (Economic growth)	State Council Framework (Collective economy)	
	Per capita annual income	SDG (Decent work)	CASS System (Per capita disposable income)	
	Private car ownership rate	WDI (Transport equipment)	State Council Framework (Car ownership rate)	
	Small agricultural machinery	WDI (Machinery)	State Council Framework (Mechanization rate)	Eleventh Schedule (Agriculture)
	Participation rate in technology training		State Council Framework (Technological advancement)	Eleventh Schedule (Technical training)
	Village enterprises/e-commerce	SDG (Economic growth)	State Council Framework (Industry)	
	Participation rate in farmers’ professional cooperatives		State Council Framework (Cooperative coverage)	
	Farmers’ professional cooperatives	SGI (Strong institution)	State Council Framework (Cooperative coverage)	
	Agricultural product trading markets	SDG (Infrastructure)	State Council Framework (Infrastructure)	Eleventh Schedule (Markets)
	Supermarkets over 50㎡	SDG (Infrastructure)	State Council Framework (Infrastructure)	Eleventh Schedule (Markets)
Habitation and Ecology	Toilet renovation rate	SDG (Sanitation)	CASS System (Sanitary toilets)	Eleventh Schedule (Sanitation)
	Centralized garbage treatment facilities	SDG (Sustainable cities)	State Council Framework (Garbage treatment)	Eleventh Schedule (Sanitation)
	Centralized sewage treatment sites	SDG (Clean water and sanitation)	State Council Framework (Sewage treatment)	Eleventh Schedule (Sanitation)
	Housing renovation rate	SGI (Environmental sustainability)	CASS System (Rural residence)	
	Clean energy (e.g., solar energy) usage rate	SGI (Environmental sustainability)	CASS System (Rural environment)	

Notes:

1. Sources reflect conceptual frameworks; identical measurement protocols were applied to all indicators in both country contexts.

2. All indicators in this table are positive indicators: higher values indicate a higher level of modernization, with all effect directions being positive.

3. Land governance indicators (e.g., small agricultural machinery, village enterprises) measure functional resource control and economic agency—not legal ownership.

4. Participation metrics: Voting rate = active electoral engagement; training participation = voluntary attendance.

5. The two road indicators — “Main village road” and “Village roads” — serve distinct purposes. Main village road evaluates external connectivity (e.g., roads linking the village to towns or markets), which is crucial for economic integration and access to regional opportunities. Village roads assess internal accessibility (e.g., streets connecting homes, schools, and clinics), reflecting intra-village mobility and equitable service distribution.

#### Entropy method.

The entropy weight method was employed to aggregate multidimensional indicators into continuous composite variables. Prior to weight calculation, all indicators are normalized to the range of [0, 1] to eliminate dimensional differences between positive and negative indicators. Weights are assigned according to the degree of information differentiation: indicators with larger cross-village variation receive higher weights, while those with limited spatial variation obtain lower weights [60–64]. Specifically, we first standardize all indicators, then calculate the proportion of each normalized value within each indicator dimension, followed by estimating the entropy value and information utility of each indicator. Finally, indicator weights are determined based on information utility, and weighted values are summed to obtain the comprehensive modernization score for each village.

The entropy-derived weights for all indicators, including overall weights and country-specific weights for China and India, are presented in Table 6. Raw data for the 32 indicators across 60 sampled villages are provided in S4 File for reproducibility.

**Table 6:** Entropy weights of village-level indicators across four dimensions.

Primary indicator	Secondary indicator	Aggregate weight			China weight			India weight		
		e_a_	d_a_	w_a_	e_c_	d_c_	w_c_	e_i_	d_i_	w_i_
Infrastructure and Public Services	Main village road	0.9663	0.0337	0.0066	0.6552	0.3448	0.0638	0.9341	0.0659	0.0093
	Village roads	0.7698	0.2302	0.0454	0.7482	0.2518	0.0466	0.9386	0.0614	0.0087
	Streetlights	0.8578	0.1422	0.0280	0.9048	0.0952	0.0176	0.7391	0.2609	0.0369
	Broadband penetration rate	0.8578	0.1422	0.0280	0.9682	0.0318	0.0059	0.6744	0.3256	0.0461
	Tap water coverage rate	0.8880	0.1120	0.0221	0.9689	0.0311	0.0057	0.7173	0.2827	0.0400
	Small-scale water conservancy facilities	0.8282	0.1718	0.0339	0.8517	0.1483	0.0274	0.8466	0.1534	0.0217
	Medical clinics/health stations	0.9324	0.0676	0.0133	0.5514	0.4486	0.0830	0.8365	0.1635	0.0231
	Coverage rate of licensed doctors per 1,000 rural residents	0.9223	0.0777	0.0153	0.9236	0.0764	0.0141	0.8913	0.1087	0.0154
Governance and Participation	Sources of public project funding	0.6652	0.3348	0.0660	0.6415	0.3585	0.0663	0.5525	0.4475	0.0634
	Village supervision methods	0.9887	0.0113	0.0022	0.8923	0.1077	0.0199	0.9807	0.0193	0.0027
	Public meeting spaces	0.8432	0.1568	0.0309	0.8391	0.1609	0.0298	0.8411	0.1589	0.0225
	Villager voting rate	0.9850	0.0150	0.0029	0.9666	0.0334	0.0062	0.9653	0.0347	0.0049
	Rate of villagers’ awareness of rights/information	0.9248	0.0752	0.0148	0.9736	0.0264	0.0049	0.8351	0.1649	0.0233
	Rural amateur cultural organizations	0.7934	0.2066	0.0407	0.8267	0.1733	0.0320	0.6461	0.3539	0.0501
	Major festival and celebration activities	0.8435	0.1565	0.0309	0.8405	0.1595	0.0295	0.8046	0.1954	0.0277
	Fitness facilities	0.8741	0.1259	0.0248	0.8912	0.1088	0.0201	0.7989	0.2011	0.0285
	Kindergartens/nurseries	0.8279	0.1721	0.0339	0.6834	0.3166	0.0586	0.8741	0.1259	0.0178
Economic Development	Annual income of village-run enterprises and collective economies	0.6185	0.3815	0.0752	0.5905	0.4095	0.0757	0.4827	0.5173	0.0732
	Per capita annual income	0.9625	0.0375	0.0074	0.9442	0.0558	0.0103	0.939	0.061	0.0086
	Private car ownership rate	0.8800	0.1200	0.0237	0.9244	0.0756	0.0140	0.8022	0.1978	0.0280
	Small agricultural machinery	0.6879	0.3121	0.0615	0.6984	0.3016	0.0558	0.6928	0.3072	0.0435
	Participation rate in technology training	0.9507	0.0493	0.0097	0.9605	0.0395	0.0073	0.8877	0.1123	0.0159
	Village enterprises/e-commerce	0.8214	0.1786	0.0352	0.9268	0.0732	0.0135	0.508	0.492	0.0697
	Participation rate in farmers’ professional cooperatives	0.8822	0.1178	0.0232	0.9242	0.0758	0.0140	0.8002	0.1998	0.0283
	Farmers’ professional cooperatives	0.8673	0.1327	0.0262	0.8641	0.1359	0.0251	0.8425	0.1575	0.0223
	Agricultural product trading markets	0.6219	0.3781	0.0745	0.7871	0.2129	0.0394	0.6508	0.3492	0.0494
	Supermarkets over 50㎡	0.7900	0.2100	0.0414	0.9051	0.0949	0.0176	0.7014	0.2986	0.0423
Habitation and Ecology	Toilet renovation rate	0.9120	0.0880	0.0174	0.6679	0.3321	0.0614	0.8793	0.1207	0.0171
	Centralized garbage treatment facilities	0.7132	0.2868	0.0566	0.4072	0.5928	0.1097	0.6273	0.3727	0.0528
	Centralized sewage treatment sites	0.6505	0.3495	0.0689	0.9605	0.0395	0.0073	0.6578	0.3422	0.0484
	Housing renovation rate	0.9507	0.0493	0.0097	0.9062	0.0938	0.0174	0.8877	0.1123	0.0159
	Clean energy (e.g., solar energy) usage rate	0.8510	0.1490	0.0294	0.6552	0.3448	0.0638	0.7004	0.2996	0.0424

Notes:

1. e: information entropy value; d: information utility value; w: weight coefficient;

2. Subscripts: a = Aggregate (China + India), c = China, i = India.

#### Variable selection and model specification.

To empirically examine how Governance and Participation, Economic Development, and Habitation and Ecology shape land-use efficiency in China and India, this study adopts ordinary least squares (OLS) regression. Given that the sample includes 60 villages and the outcome variable is a continuous welfare indicator, OLS is an appropriate and robust estimator.

Following Ostrom’s CPR theory, rural land-use efficiency is conceptualized as the ultimate welfare outcome of governance effectiveness. Therefore, rural living satisfaction is employed as the proxy (instrumental variable) for land-use efficiency, rather than the entropy-weighted infrastructure index, to avoid endogenous correlation and inflated goodness-of-fit. To mitigate multicollinearity among Governance and Participation, Economic Development, and Habitation and Ecology, principal component analysis (PCA) is used to construct a unified core capacity factor. As presented in Table 7, the first principal component yields an eigenvalue of 3.012 and accounts for 94.66% of total variance. Factor loadings for the three dimensions are 0.981, 0.989, and 0.957 respectively, all exceeding 0.95, indicating strong representation of each dimension. Therefore, the first component is adopted as the core capacity factor to capture comprehensive village‑level development capacity.

**Table 7:** PCA results.

Factor	Loading	Eigenvalue	Variance
Governance and Participation	0.981	3.012	94.66%
Economic Development	0.989		
Habitation and Ecology	0.957		

The regression analysis includes three nested specifications: baseline model, moderation model, and mediation model. Control variables consist of village type, per capita arable land (log-transformed), and village altitude (log-transformed), along with a country dummy variable.

(1) Baseline model:

$$\begin{array}{c} {Satisfaction}_{i}=\alpha +\beta_{\text{1}\;}{Corefactor}_{i}\;+\beta_{\text{2}\;}{Country}_{i}+\beta_{\text{3}\;}{Villagetype}_{i}{+\beta_{\text{4}\;}{Land}_{i}+\beta }_{\text{5}\;}{Altitude}_{i\;}+\in_{i} \end{array}$$

where ${Satisfaction}_{i}$ denotes rural living satisfaction (proxy for land-use efficiency); ${Corefactor}_{i}$ is the composite core capacity factor derived from PCA; ${Country}_{i}$, ${Villagetype}_{i}$, ${Land}_{i}$, and ${Altitude}_{i\;}$ represent the country dummy, village type dummy, log per capita arable land, and log altitude, respectively; α is the intercept; $\beta_{\text{1}\;}$–$\beta_{\text{5}\;}$ are regression coefficients; and $\in_{i}$ is the random error term.

(2) Moderation model:

$$\begin{array}{c} {Satisfaction}_{i}=\alpha +\beta_{\text{1}\;}{Corefactor}_{i}\;+\beta_{\text{2}\;}{Country}_{i}+\beta_{\text{3}\;}(Corefactor\times Country)i+controls+\in_{i} \end{array}$$

where Corefactor×Country is the interaction term testing the moderating effect of national institutional context; controls include village type, per capita arable land, and altitude.

(3) Mediation model:

$$\begin{array}{c} {Infrustructure}_{i}=\alpha +a\cdot {Corefactor}_{i}\;+controls+\in_{i} \end{array}$$ (a)

$$\begin{array}{c} {Satisfaction}_{i}=\alpha +b\cdot {Infrustracture}_{i}+c{\;}^{\prime }\cdot {Corefactor}_{i}\;+controls+\in_{i} \end{array}$$ (b)

where ${Infrustructure}_{i}$ is the entropy-weighted score of Infrastructure and Public Services; a is the coefficient of core capacity on infrastructure; b is the coefficient of infrastructure on satisfaction; and $c{\;}^{\prime }$ is the direct effect of core capacity after controlling for the mediating variable. Multicollinearity, heteroscedasticity, and a series of robustness checks are conducted to ensure the reliability of the estimation results. All raw data supporting the regression models are available in S4 and S5 Files for reproducibility.

Table 8 reports the descriptive statistics for all variables used in the empirical analysis. The sample includes 60 villages, evenly distributed between China and India, with 35% located in urban–rural fringe zones and 65% classified as agricultural villages. The dependent variable, rural living satisfaction ranges from 0.167 to 1.000, with a mean of 0.662, indicating moderate to high rural welfare and substantial cross-village variation. The core capacity factor, standardized to a mean of 0 and standard deviation of 1, shows considerable dispersion, reflecting marked differences in village core capacity across villages. Infrastructure and Public Service exhibits relatively low but stable values, consistent with standardized indicator properties. Geographic and institutional controls—including per capita arable land, altitude, national institutional context, and tap water access—display sufficient variation for regression estimation. No outliers or irregular distributions are detected, ensuring a robust foundation for cross-national comparative analysis.

**Table 8:** Descriptive statistics.

Variable	Obs	Mean	Std.Dev	Min	Max
Satisfaction	60	0.662	0.225	0.167	1.000
CoreFactor	60	0.000	1.000	−2.123	2.654
Infrastructure	60	0.048	0.026	0.010	0.116
Country	60	0.500	0.504	0	1
VillageType	60	0.350	0.481	0	1
LnLand	60	0.815	0.526	−1.435	2.078
LnAlt	60	5.012	1.326	2.102	7.895
TapWater	60	0.650	0.481	0	1

Note: Country dummy: 1 = China, 0 = India; Village type dummy: 1 = Urban–rural fringe zone, 0 = Agricultural village; Tap water dummy: 1 = With access, 0 = Without access.

Table 9 presents the pairwise Pearson correlation coefficients among all variables. Rural living satisfaction is strongly and positively correlated with the core capacity factor (r = 0.512, p < 0.01), providing preliminary evidence that higher core capacity is associated with greater land-use efficiency. Infrastructure and Public Service is also significantly correlated with satisfaction (r = 0.436, p < 0.01), consistent with the expectation that improved public goods provision enhances rural welfare. In addition, the strong positive association between the core capacity factor and Infrastructure and Public Service (r = 0.821, p < 0.01) supports the hypothesized mediating pathway. National institutional context is positively correlated with satisfaction (r = 0.228, p < 0.05), indicating the presence of cross-country variation. By contrast, per capita arable land is negatively associated with satisfaction (r = −0.145, p < 0.1), suggesting that villages with relatively scarce land resources may achieve higher levels of efficiency. Importantly, all pairwise correlation coefficients are below 0.9, indicating the absence of severe multicollinearity and supporting the appropriateness of OLS estimation.

**Table 9:** Correlation matrix.

Variables	(1)	(2)	(3)	(4)	(5)	(6)	(7)	(8)
(1) Satisfaction	1.000							
(2) CoreFactor	0.512***	1.000						
(3) Infrastructure	0.436***	0.821***	1.000					
(4) Country	0.228**	0.165*	0.198**	1.000				
(5) VillageType	0.174*	0.132	0.145	0.089	1.000			
(6) LnLand	−0.145*	−0.112	−0.098	0.065	0.071	1.000		
(7) LnAlt	−0.092	−0.081	−0.072	0.108	0.053	0.119	1.000	
(8) TapWater	0.183**	0.169*	0.211**	0.242**	0.097	0.051	−0.068	1.000

Note: *** p < 0.01, ** p < 0.05, * p < 0.1.

To further assess potential multicollinearity, variance inflation factors (VIF) were calculated for all explanatory variables. The results indicate that all VIF values are below 2.1, while the corresponding tolerance values (1/VIF) exceed 0.48. These values are well within both the conventional threshold of 5 and the more conservative benchmark of 3. The highest VIF values are observed for the interaction term between the core capacity factor and national institutional context (VIF = 2.07) and for the core capacity factor itself (VIF = 2.03). All control variables exhibit low VIF values. Overall, these findings suggest that multicollinearity is not a serious concern, thereby supporting the reliability and stability of the regression estimates.

## Results

### Entropy-based analysis of rural modernization

The entropy weight method was applied to 32 indicators across 60 sampled villages in China and India to construct a composite index of rural modernization. By assigning weights according to cross-village variability, the method ensures that indicators with greater discriminatory power contribute more substantially to the overall score.

Across the full sample (see Table 6), the indicators with the highest aggregate weights include agricultural product trading markets (w_a_ = 0.0745), annual income of village-run enterprises and collective economies (w_a_ = 0.0752), sources of public project funding (w_a_ = 0.0660), small agricultural machinery (w_a_ = 0.0615), and centralized sewage treatment sites (w_a_ = 0.0689). These indicators exhibit relatively high information utility values, indicating strong cross-village variation and a substantial contribution to the composite modernization index. In contrast, several governance-related indicators such as village supervision methods (w_a_ = 0.0022; e_a_ = 0.9887) and villager voting rate (w_a_ = 0.0029; e_a_ = 0.9850) display entropy values close to one, reflecting limited variability and consequently low weight contributions within the index construction.

A comparison of entropy-derived weights between China and India reveals notable differences in indicator dispersion patterns. In the Chinese subsample, relatively high weights are observed for centralized garbage treatment facilities (w_c_ = 0.1097), medical clinics and health stations (w_c_ = 0.0830), annual income of village-run enterprises and collective economies (w_c_ = 0.0757), and small agricultural machinery (w_c_ = 0.0558). In the Indian subsample, higher weights are concentrated in village enterprises and e-commerce (w_i_ = 0.0697), annual income of village-run enterprises and collective economies (w_i_ = 0.0732), agricultural product trading markets (w_i_ = 0.0494), broadband penetration rate (w_i_ = 0.0461), and centralized sewage treatment sites (w_i_ = 0.0484). These differences reflect variation in cross-village dispersion within each national sample.

The comprehensive scores and rank for all selected villages are listed in Table 10, with country-stratified results provided in S6 and S7 Files. The entropy-based comprehensive modernization scores for the 60 villages range from 0.0391 to 0.4748, with a mean value of 0.1787 and a positive skewness of 0.98. This distribution indicates that most villages cluster at relatively low modernization levels, while a smaller number achieve substantially higher scores. When disaggregated by country, Chinese villages (Village IDs 1–30) record a mean score of 0.2520 with a standard deviation of 0.0761, whereas Indian villages (Village IDs 31–60) exhibit a mean of 0.1055 and a standard deviation of 0.0330. The average modernization level of Chinese villages in the sample is therefore more than twice that of Indian villages. At the upper end of the distribution, the highest-scoring village is Indian (0.4748), while the lowest score in the full sample (0.0391) is also observed in India, indicating greater dispersion at both extremes within the Indian subsample.

**Table 10:** Comprehensive scores and rank for the villages surveyed.

Rank	Village ID	Comprehensive score	Rank	Village ID	Comprehensive score
1	39	0.474795213	31	42	0.167398416
2	37	0.446567756	32	7	0.163776728
3	35	0.407136611	33	14	0.155929608
4	40	0.331745187	34	13	0.150481486
5	23	0.314115096	35	20	0.148625469
6	10	0.298502004	36	17	0.146047131
7	47	0.288846244	37	57	0.144506125
8	1	0.278654265	38	34	0.144314927
9	27	0.275407169	39	6	0.129590286
10	36	0.26093369	40	2	0.127752538
11	26	0.255702135	41	60	0.126091812
12	8	0.248834558	42	45	0.125559074
13	38	0.241738173	43	55	0.114452622
14	29	0.233072813	44	43	0.109201087
15	25	0.226732131	45	50	0.104332715
16	19	0.223229188	46	52	0.103397018
17	24	0.222357589	47	21	0.10213321
18	9	0.22018984	48	53	0.097968305
19	28	0.209412891	49	48	0.096254439
20	30	0.207998399	50	59	0.080604802
21	46	0.203893588	51	54	0.078935112
22	4	0.203008194	52	44	0.076748099
23	16	0.201333843	53	33	0.073654729
24	15	0.198504082	54	41	0.071010862
25	11	0.186070113	55	49	0.06177329
26	18	0.184351583	56	32	0.058966694
27	12	0.182663721	57	56	0.057458171
28	5	0.180305369	58	51	0.055746565
29	22	0.178561938	59	58	0.05209032
30	3	0.17442466	60	31	0.039123159

Note: Village IDs 1–30 China, and village IDs 31–60 India.

The ranking of villages further illustrates this distributional pattern. The top five villages record comprehensive scores of 0.4748, 0.4466, 0.4071, 0.3317, and 0.3141, respectively, while the bottom five villages record scores of 0.0590, 0.0575, 0.0557, 0.0521, and 0.0391. Notably, four of the top five villages are from India, and all of the bottom five villages also belong to India, indicating an extremely high level of within-country disparity in rural modernization among Indian villages. A substantial number of villages cluster within the intermediate range between approximately 0.150 and 0.300, indicating moderate levels of modernization across a significant portion of the sample.

Geographic differentiation also reveals variation in modernization outcomes. In China, villages located in beach areas record the highest average comprehensive score (0.2415), followed by mountainous areas (0.2200), plains (0.1864), and hills (0.2036). In India, villages in hilly areas record the highest average score (0.3537), followed by reservoir areas (0.2765), while plains exhibit the lowest average score (0.0876). These results indicate substantial intra-country variation across terrain types.

Regional comparisons further demonstrate heterogeneity in modernization levels. Among the selected Chinese provinces, Gansu records a comprehensive score of 0.2280, exceeding Chongqing (0.2050) and Shanxi (0.1899). In India, Tamil Nadu achieves a comprehensive score of 0.2629, followed by Punjab (0.1447), while Bihar records 0.0618. These figures indicate substantial regional dispersion within both national samples. These patterns invite further regional analysis for policy actors.

Village-type classification provides additional differentiation. In China, agricultural villages record an average score of 0.2015, while urban–rural fringe villages reach 0.2111. In India, agricultural villages average 0.1457, whereas urban–rural fringe villages record 0.2555. In both countries, urban–rural fringe villages display higher modernization scores than purely agricultural villages.

Finally, comparison between GDP levels and modernization outcomes suggests that the relationship is not strictly linear within the sampled regions, particularly in China (see Table 11). In China, Gansu (low GDP) records a modernization score of 0.2280, exceeding Shanxi (middle GDP, 0.1899) and Chongqing (high GDP, 0.2050). In India, Tamil Nadu (high GDP) achieves 0.2629, while Punjab (middle GDP) records 0.1447 and Bihar (low GDP) records 0.0618. These descriptive patterns indicate that GDP level alone does not correspond proportionally to modernization scores in the sample.

**Table 11:** Relationship between GDP levels and village modernization scores.

GDP Level	Representative province in China	Representative state in India	Correlation between modernization score and GDP
High	Chongqing (¥87,000 per capita)	Tamil Nadu (₹212,174 per capita)	Positive correlation (Tamil Nadu scores the highest), but Chongqing lags in habitation and ecology governance relative to its economic level.
Middle	Shanxi (¥64,700 per capita)	Punjab (₹149,894 per capita)	Weak correlation (Shanxi scores 0.1899, lower than Gansu’s 0.2280), reflecting the challenges of resource-based economic transformation.
Low	Gansu (¥40,900 per capita)	Bihar (₹43,605 per capita)	Negative correlation (Gansu outperforms expectations), with policy support (e.g., Western Development Strategy) offsetting GDP disadvantages.

### Baseline regression and institutional moderation

The OLS estimates for the baseline and institutional moderation models are reported in Table 12. Column (1) presents the baseline specification including only the control variables. The results show that national institutional context, village type, and per capita arable land are statistically significant. Specifically, villages in China and those located in urban-rural fringe areas in either country exhibit higher levels of land-use efficiency, and villages with relatively scarce arable land also tend to achieve greater efficiency. Column (2) introduces the core capacity factor. The coefficient is positive and statistically significant at the 1% level (β = 0.092, p < 0.01), indicating that stronger core capacity significantly enhances land-use efficiency. Column (3) further incorporates the interaction term between core capacity factor and national institutional context to examine the moderating role of institutions. The interaction term is positive and significant (β = 0.017, p < 0.05), suggesting that China’s centralized governance structure strengthens the positive relationship between core capacity factor and land-use efficiency. Meanwhile, the explanatory power of the model increases substantially, with R² rising from 0.312 in the baseline model to 0.453 in the moderation model. Overall, these findings provide strong support for the proposed hypotheses and are consistent with the theoretical expectations of the study.

**Table 12:** Regression results.

Variables	(1) Baseline	(2) Core Factor	(3) Moderation
CoreFactor	—	0.092*** (0.034)	0.104*** (0.038)
Country	0.124** (0.061)	0.097* (0.057)	0.102** (0.050)
CoreFactor×Country	—	—	0.017** (0.008)
VillageType	0.098** (0.048)	0.076* (0.045)	0.074* (0.044)
LnLand	−0.071* (0.042)	−0.062* (0.037)	−0.060* (0.036)
LnAlt	−0.046 (0.035)	−0.039 (0.032)	−0.037 (0.031)
Constant	0.685*** (0.079)	0.661*** (0.076)	0.658*** (0.074)
N	60	60	60
R²	0.312	0.447	0.453

Note: *** p < 0.01, ** p < 0.05, * p < 0.1. Robust standard errors in parentheses.

### Heterogeneity analysis across national institutional contexts

To further examine whether the relationship between village core capacity and land-use efficiency varies across national institutional contexts, the full sample was divided into Chinese and Indian subsamples for heterogeneity analysis. The results, presented in Table 13, reveal that the core capacity factor exerts a consistently positive effect on land-use efficiency in both countries, although the magnitude of the effect differs across national institutional contexts.

**Table 13:** Heterogeneity analysis.

Variables	(1) China	(2) India
CoreFactor	0.106*** (0.037)	0.078** (0.037)
VillageType	0.071* (0.041)	0.067* (0.039)
LnLand	−0.058* (0.034)	−0.064* (0.037)
LnAlt	−0.035 (0.033)	−0.041 (0.036)
Constant	0.655*** (0.083)	0.642*** (0.086)
N	30	30
R²	0.421	0.386

Notes: *** p < 0.01, ** p < 0.05, * p < 0.1. Robust standard errors in parentheses.

In the Chinese subsample (Column 1), the coefficient of the core capacity factor is positive and statistically significant at the 1% level (β = 0.106, p < 0.01), with relatively stronger explanatory power (R² = 0.421) than in the Indian subsample. In the Indian subsample (Column 2), the effect remains positive but is only significant at the 5% level (β = 0.078, p < 0.05), accompanied by a slightly lower model fit (R² = 0.386). These results are consistent with the moderating effect identified in the full-sample regression analysis. Specifically, China’s centralized national institutional context appears to better facilitate the translation of core capacity into land-use efficiency, whereas India’s comparatively decentralized governance system may generate higher coordination costs and institutional frictions, thereby weakening the marginal effect of villages’ core capacity.

Across both subsamples, village type and per capita arable land display consistent signs and significance levels, suggesting that the effects of spatial location and land scarcity remain stable regardless of national institutional context. Altitude is statistically insignificant in both models, indicating no robust linear relationship between geographic elevation and land-use efficiency in either country. Taken together, these findings demonstrate that the positive effect of village core capacity on land-use efficiency is both statistically robust and institutionally contingent, with stronger effects observed under more centralized governance structures.

### Mediating role of infrastructure provision

Table 14 presents the results of the mediation analysis examining whether Infrastructure and Public Service mediates the relationship between village core capacity and land-use efficiency. Column (1) reports the total effect model, in which the core capacity factor is positively and significantly associated with land-use efficiency (β = 0.092, p < 0.01). Column (2) estimates the first-stage mediation model and shows that the core capacity factor significantly improves Infrastructure and Public Service (β = 0.078, p < 0.01). Column (3) presents the final mediation model including both the core capacity factor and Infrastructure and Public Service. The coefficient of the core capacity factor remains positive and statistically significant (β = 0.061, p < 0.05), while Infrastructure and Public Service also exerts a positive and highly significant effect on land-use efficiency (β = 0.083, p < 0.01). The reduction in the coefficient magnitude of the core capacity factor after the inclusion of Infrastructure and Public Service indicates the presence of a partial mediation effect. This suggests that Infrastructure and Public Service constitutes an important mechanism through which village core capacity factor enhances land-use efficiency. In addition, model fit improves after the inclusion of the mediator, with R² increasing from 0.447 in the total-effect model to 0.476 in the final model.

**Table 14:** Mediation analysis of Infrastructure and Public Services in the relationship between core capacity and land-use efficiency.

Variables	(1) Total Effect	(2) Infrastructure and Public Service	(3) Final Model
CoreFactor	0.092*** (0.034)	0.078*** (0.029)	0.061** (0.030)
Infrastructure and Public Service	—	—	0.083*** (0.030)
Constant	0.661*** (0.076)	0.043*** (0.015)	0.624*** (0.072)
Controls	Yes	Yes	Yes
R²	0.447	0.412	0.476

Notes: *** p < 0.01, ** p < 0.05, * p < 0.1. Robust standard errors are used. Control variables include village type, per capita arable land, altitude, etc.

### Robustness checks

Table 15 reports the results of a series of robustness checks designed to verify the stability and reliability of the main findings. Column (1) presents the baseline model for comparison, in which the core capacity factor remains positively and significantly associated with land-use efficiency at the 1% level (β = 0.092, p < 0.01). Column (2) applies a modest 1% and 99% winsorization (setting more extreme values to these limits) to reduce the potential influence of extreme observations. The coefficient of the core capacity factor remains highly significant and largely unchanged in magnitude (β = 0.089, p < 0.01), suggesting that the results are not driven by outliers. Column (3) introduces access to tap water access as an additional control variable to address potential omitted-variable bias. The estimated effect of the core capacity factor remains stable and statistically significant (β = 0.090, p < 0.01). Column (4) restricts the sample to agricultural villages only. The core capacity factor continues to exhibit a significantly positive effect (β = 0.085, p < 0.01), indicating that the main findings are not limited to urban–rural fringe villages and remain valid within more traditional agricultural settings. Column (5) replaces the OLS specification with a Probit model and reports average marginal effects. The coefficient of the core capacity factor remains positive and significant at the 1% level (β = 0.031, p < 0.01), demonstrating that the results are robust to alternative model specification.

**Table 15:** Robustness checks.

Variables	(1) Baseline	(2) Winsor 1%	(3) Add Tap Water	(4) Subsample Agricultural	(5) Probit
CoreFactor	0.092*** (0.034)	0.089*** (0.033)	0.090*** (0.034)	0.085*** (0.031)	0.031*** (0.011)
Country	0.097* (0.057)	0.094* (0.056)	0.092* (0.055)	0.089** (0.044)	0.037** (0.018)
VillageType	0.076* (0.045)	0.074* (0.044)	0.072* (0.043)	—	0.029* (0.017)
LnLand	−0.062* (0.037)	−0.059* (0.036)	−0.060* (0.036)	−0.057* (0.033)	−0.022* (0.013)
LnAlt	−0.039 (0.032)	−0.037 (0.031)	−0.038 (0.031)	−0.035 (0.030)	−0.014 (0.012)
TapWater	—	—	0.024 (0.023)	—	—
Constant	0.661*** (0.076)	0.657*** (0.075)	0.659*** (0.075)	0.648*** (0.077)	0.671*** (0.088)
N	60	60	60	39	60
R²/ Pseudo R²	0.447	0.451	0.454	0.426	0.297

Notes: *** p < 0.01, ** p < 0.05, * p < 0.1. Robust standard errors are reported in parentheses.

Across all robustness tests, the control variables maintain signs and significance levels broadly consistent with those reported in the baseline model, and no abnormal fluctuations in model fit are observed. Overall, these findings confirm that the positive effect of village core capacity on land-use efficiency is highly robust and not driven by extreme values, omitted variables, sample selection, or model specification choices.

## Discussion

This study investigates how village-level core capacity, national institutional context, and spatial characteristics shape land-use efficiency across rural villages in China and India. Drawing upon CPR theory and the IAD framework, the empirical analysis provides strong support for the proposed hypotheses and reveals substantial cross-national heterogeneity in rural modernization pathways. The findings suggest that rural modernization is shaped not only by economic development or infrastructure investment alone, but more fundamentally by the institutional capacity to coordinate collective action, allocate public resources, and reduce transaction costs within different governance systems.

First, the core capacity factor—which integrates Governance and Participation, Economic Development, and Habitation and Ecology—has a significant positive effect on land-use efficiency (instrumentally proxied by rural living satisfaction), as predicted by H1. This finding is highly consistent with the central proposition of Ostrom’s CPR theory, which emphasizes that effective collective governance can reduce transaction costs, strengthen monitoring and compliance, and promote the sustainable management of shared resources such as land. Even after controlling for national institutional context, village type, per capita arable land, altitude, and other variables, the positive effect remains stable and robust. This suggests that core capacity involves universal and critical determinants of rural land-use efficiency.

Second, the national institutional context plays a significant moderating role in shaping the relationship between core capacity and land-use efficiency. The positive effect of core capacity is significantly stronger under China’s centralized governance system than under India’s more decentralized institutional environment, thereby supporting H2 which predicted differential outcomes mediated by differences in national institutional contexts. Importantly, this does not imply that one institutional model is inherently superior to the other. Rather, the findings indicate that macro-institutional arrangements influence the extent to which village core capacity can be effectively translated into development outcomes. China’s centralized governance structure appears to reduce land transaction costs and interdepartmental coordination costs, thereby facilitating collective action and policy implementation. By contrast, India’s decentralized governance framework is more vulnerable to fragmented land property rights, uneven fiscal capacity, and higher coordination costs, which together on average weaken the consistency and effectiveness of collective action in improving land-use efficiency while also generating a broader range of outcomes at the extremes.

Third, urban-rural fringe villages demonstrate significantly higher land-use efficiency than traditional agricultural villages, and this advantage becomes more pronounced as per capita arable land decreases as predicted by H3. Urban-rural fringe villages are typically exposed to stronger land scarcity pressures and more diversified economic activities, which encourage more intensive land use, more efficient spatial planning, and better infrastructure provision. In contrast, remote agricultural villages tend to rely on relatively extensive land-use patterns, while both their incentives and capacities for improving land-use efficiency remain comparatively limited. This finding highlights the joint role of spatial location and resource endowment in shaping governance incentives and land-use outcomes.

The descriptive findings further reveal substantial within-country heterogeneity, particularly in India, suggesting that national institutional arrangements alone cannot fully explain rural modernization outcomes. The comprehensive modernization score of Tamil Nadu (0.2629) is nearly double that of Punjab (0.1447), the second-highest performing state, while Bihar registers the lowest score at only 0.0618. This striking divergence cannot be fully explained by terrain alone: Tamil Nadu includes hilly and reservoir areas, whereas Punjab and Bihar are predominantly plain terrain favorable for agriculture. Instead, the key lies in how terrain interacts with local governance and economic conditions. Complex topography in Tamil Nadu has encouraged stronger collective governance and coordinated infrastructure planning, which support higher land-use efficiency. By contrast, the flat plains of Punjab and Bihar have not automatically translated into better modernization outcomes. In particular, Bihar’s weak institutional capacity and geographical constraints, such as frequent flooding and poor rural connectivity, severely limit effective land utilization, resulting in the lowest regional performance. At the village level, India also displays extreme polarization: four of the five highest-performing villages are from India, while all five of the lowest-performing villages also belong to India. This dual pattern of outstanding top performers and lagging villages concentrated in India reinforces strong within-country heterogeneity that goes beyond national institutional design alone.

The heterogeneity analysis demonstrates that the core capacity factor exerts a significantly positive effect in both Chinese and Indian subsamples, although the magnitude and significance of the coefficient are notably greater in China. This result is fully consistent with the moderating effect identified in the full-sample regression analysis. In addition, the mediation analysis confirms that Infrastructure and Public Service plays a significant partial mediating role. Specifically, core capacity enhances land-use efficiency both directly and indirectly through improvements in Infrastructure and Public Service. This finding indicates that Infrastructure and Public Service constitutes an important transmission channel linking governance capacity to development outcomes. Furthermore, the robustness tests show that the main results remain stable after controlling for extreme values, adding additional control variables, conducting subgroup regressions, and varying the functional form of the estimation model. Collectively, these results demonstrate the reliability and robustness of the study’s conclusions.

From a theoretical perspective, this study extends CPR theory beyond its traditional focus on localized micro-level cases and demonstrates its continued explanatory power in cross-national comparative research on rural modernization. Methodologically, the study also addresses the problems of multicollinearity and homologous fitting bias that commonly arise in entropy-weight-based evaluation systems by combining PCA with instrumental proxy variables. This provides a useful methodological reference for future studies employing multidimensional evaluation frameworks. From a practical perspective, the findings clarify the synergistic relationships among governance, economic development, national institutional contexts, and spatial characteristics, thereby offering a more comprehensive analytical framework for understanding the differentiated pathways of rural modernization.

## Conclusion

Based on village-level survey data from China and India, this study systematically explores the multidimensional drivers of rural land-use efficiency under cross-national institutional differences. Synthesizing the empirical results, four core conclusions are summarized.

First, village core capacity serves as a universal positive driver of land-use efficiency. Second, Infrastructure and Public Service acts as a critical partial mediator linking core capacity to land-use efficiency. Third, national institutional context exerts a notable moderating effect, with centralized governance facilitating more effective capacity transformation than decentralized systems. Fourth, spatial location and land scarcity jointly shape land-use efficiency, with urban-rural fringe villages outperforming remote agricultural villages. Overall, rural land-use efficiency improvement relies on the synergistic coordination of governance, economy development, national institutional contexts, and spatial characteristics, rather than single-factor dominance.

Theoretically, this study expands the application boundary of CPR theory to macro‑level cross‑national rural comparative research. Methodologically, it optimizes multidimensional indicator evaluation by integrating PCA and independent outcome proxies, resolving common empirical defects in entropy‑weighted analysis and providing replicable references for similar developing‑economy studies.

Beyond context-specific findings, this study delivers generalized lessons for global rural modernization. Rural land governance effectiveness depends not merely on economic development but on institutional compatibility that aligns grassroots governance, public participation, and resource allocation mechanisms. Institutional decentralization or centralization cannot be judged in isolation; their efficiency gains hinge on whether institutional arrangements reduce transaction costs, resolve coordination frictions, and adapt to local resource endowments. In this sense, sustainable rural transformation in developing economies requires balanced coordination between infrastructural and public service, governance nad participation, economic development, and ecological management, rather than one-dimensional modernization strategies.

### Policy implications

Drawing on the empirical findings, this study puts forward targeted, differentiated, and context-sensitive policy implications for China, India, and other developing economies to advance rural land-use efficiency and inclusive rural welfare.

For developing economies, priority should be given to enhancing grassroots governance capacity, expanding public participation, and improving multi-level supervision mechanisms, so as to lower transaction costs in land resource allocation and public goods provision. It is essential to strengthen coordination between grassroots governance and local economic development by channeling public investment into capacity building, infrastructure construction, and ecological management, which can reconcile short-term economic expansion with long-term sustainable land use. In addition, spatially differentiated governance strategies are required: urban-rural fringe villages should capitalize on locational advantages to promote intensive and compact land utilization; by contrast, remote agricultural villages need increased investment in infrastructure and ecological governance to narrow efficiency gaps and improve equity.

At the national level, country-specific institutional adjustments are needed. China should maintain the coordination advantages of its centralized governance system while scaling up balanced public service investment to mitigate regional disparities in rural land governance outcomes. Grassroots land governance experiments advance integrated rural infrastructure development through consolidated village land management. Local practices and empirical outcomes confirm that unified land planning paired with road, water supply and sanitation infrastructure substantially raises land-use efficiency and villagers’ living satisfaction. Drawing on these bottom-up grassroots insights, the central government has formalized such village-scale infrastructure governance models into national rural development policies, supporting more targeted fiscal resource allocation and higher land-use efficiency nationwide. India needs to mitigate institutional fragmentation, clarify land property rights, strengthen fiscal transfer mechanisms and cross-regional coordination, and improve policy implementation capacity within its decentralized governance framework.

More generally, developing economies should avoid blind institutional imitation and one-size-fits-all policy copying. Instead, they should pursue context-adapted institutional compatibility and cross-sectoral policy synergy. Ultimately, sustainable rural modernization and efficient land governance depend on the integrated coordination of grassroots governance, economic development, national institutional context, and spatial characteristics.

### Study limitations

Several limitations should be acknowledged when interpreting the findings of this study. First, regarding indicator construction, although the entropy‑weighted method reduces subjective bias, composite indicators are still sensitive to indicator selection and cross‑village variance. Dimensions with low statistical variation may be underweighted despite their structural importance to rural modernization. Second, inconsistent administrative classification and data reporting standards between China and India may weaken cross‑national comparability. Third, the cross‑sectional research design limits causal identification. While this study adopts robustness checks, omitted variable bias, measurement errors, and unobserved village‑level heterogeneity cannot be fully eliminated, leaving potential endogeneity risks unresolved. Fourth, the sample of 60 villages restricts generalizability. Data collection in 2021 coincided with the normalization phase of COVID-19, which may have constrained the depth of household surveys in remote regions. Rural living satisfaction, though theoretically valid, is an indirect instrumental proxy for land‑use efficiency; future research could incorporate direct indicators of land productivity and ecological sustainability. In addition, this study measures land‑use rights via functional control rather than formal ownership, neglecting informal tenure and customary land practices that shape real‑world land governance outcomes which could also influence rural living satisfaction. Finally, the absence of longitudinal data prevents the analysis of long‑term dynamic effects. Future research could expand the geographic and temporal scope, adopt panel data or mixed‑method designs, and explore the evolution of rural governance under rapid urbanization and climate change, to strengthen causal inference and deepen the understanding of rural modernization trajectories.

## Supporting information

S1 File Leader and villager questionnaires. (DOCX)

S2 File Questionnaire data for Chinese villages. (XLSX)

S3 File Questionnaire data for Indian villages. (XLSX)

S4 File Raw data for entropy method. (XLSX)

S5 File Raw data for OLS. (XLSX)

S6 File Comprehensive scores and rank for Chinese villages. (XLSX)

S7 File Comprehensive scores and rank for Indian villages. (XLSX)
