# Supplementary material for: Rural governance capacity and land-use efficiency: A comparative analysis of rural modernization in China and India
Source: PLoS One. 2026 Aug 25;21(8):e0356874. doi: 10.1371/journal.pone.0356874 (PMC13505948; doi:10.1371/journal.pone.0356874)
Supplement: Supplementary file 1 [file pone.0356874.s001.docx]

**Leader Questionnaires for Rural Public Goods**

**Part A Basic Information of the Village**

_____village_____(Tehsil/Tahsil/Taluka/Block/Mandal)_____(District/Division)_____State

A1. Which type of your village is?

Options:

Agricultural

Urban-rural fringe zone

Town government located in

Town government located in and Urban-rural fringe zone

A2. The geography of the village is?

Options:

Plain

Hills

Mountains

Reservoir area

Beach area

Others

A3. The total households of the village are ____, the whole population is ____, men are ____, the people with migrant worker experience or doing business in cities are ____.

A4. The average annual income per capita of the village:

2018: ____

2019: ____

2020: ____

A5. The total arable land of the village is ____ hectares, the total woodland is ____ hectares.

A6. Is there any villager-run enterprise or collective economy?

Options:

Yes

No (Skip to A8)

A7. If yes, the number of workers is ____, the annual output value is ____, the net profit is ____.

A8. Which type of the village road to block panchayat is?

Options:

Muddy soil road (Skip to A10)

Gravel road (Skip to A10)

Tar road

Cement road

A9. The village road to block/district is built in ____ (year).

A10. The whole hardened village roads are ____ meters, the road lamps are ____ (if no hardened roads, please write "0").

A11. Is there tap water in the village?

Options:

Yes

No (Skip to A13)

A12. The first tap water was installed in ____ (year), ____ (number) of households have already got the tap water.

A13. The electricity was firstly connected in ____ (year), which was transformed in ____ (year), the electricity fee standard of the village (2021) is ____ /kW·h.

A14. The first fixed telephone of the village was installed in ____ (year), ____ (number) of households have got the fixed telephone nowadays.

A15. The first cable television of the village was available in ____ (year), ____ (number) of households have already got the cable service.

A16. The first broadband Internet was available in the village in ____ (year), ____ (number) of households have already access to broadband Internet.

A17. Is there any centralized treatment or partially centralized treatment of household garbages in the village?

Options:

Yes (Please write down the total number on the line)

No

A18. Is there any centralized sewage treatment or partial centralized treatment in the village?

Options:

Yes (Please write down the total number on the line)

No

A19. Has the village achieved or partially finished the toilet revolution?

Options:

Yes (Please write down the number of households finished toilet revolution on the line)

No

A20. Is there any kindergarten or nursery in the village?

Options:

Yes (Please write down the total number on the line)

No

A21. Is there any sports and fitness place in this village?

Options:

Yes (Please write down the total number on the line)

No

A22. Is there any temple in the village?

Options:

Yes (Please write down the total number on the line)

No

A23. Is there any farmers' amateur cultural organizations?

Options:

Yes (Please write down the total number on the line)

No

A24. Does the village regularly hold any collective celebration for festivals?

Options:

Yes (Please write down times per year to hold such celebrations on the line)

No

A25. Is there any medical clinic in the village?

Options:

Yes (Please write down the total number on the line)

No

A26. Is there any practicing (assistant) physician in the village?

Options:

Yes (Please write down the total number on the line)

No

A27. Is there agricultural machinery in the village (tractor, seeder, motorized threshing machine, etc.)?

Options:

Yes (Please write down the total number on the line)

No

A28. Is there any canal/pond/reservoir available for irrigation?

Options:

Yes (Please write down the total number on the line)

No

A29. What's the main source of irrigation water in the village?

Options:

Surface water

Underground water

A30. Is there any specialized cooperative established by villagers in the village, such as self-help group, cooperative for better farming?

Options:

Yes (Please write down the total number on the line)

No

A31. Is there any agricultural products trading market in this village?

Options:

Yes (Please write down the total number on the line)

No

A32. Is there any comprehensive store or supermarket with more than 50 m² in this village?

Options:

Yes (Please write down the total number on the line)

No

A33. The funds of public welfare projects (roads, reservoirs, irrigation facilities, etc.) mainly come from ____.

Options:

The central and local governments

The surplus of the village collective economy

Donation of villagers

Loans from national or regional banks

Donation of enterprises

Donation of NGOs

Others (Please write down on the line)

A34. Who is responsible for supervising the expenditure of public welfare projects?

Options:

The village panchayat

The block/district authorities

Make it public to villagers

No supervision

I'm not sure

A35. Is there any gathering place or hall for all villagers to discuss village affairs?

Options:

Yes (Please write down the total number on the line)

No

**Villager Questionnaires for Rural Public Goods**

**Part B Satisfaction with Public Goods Provision in the Village**

_____village_____(Tehsil/Tahsil/Taluka/Block/Mandal)_____(District/Division)_____State

**B Basic Information of the Villager Surveyed**

B1. Your gender

Options: 1. Male 2. Female

B2. Your degree of education

Options: 1. Below primary school 2. Junior middle school 3. High school 4. College or above

B3. Your age:

Filled content: 37

B4. Your marital status

Options: 1. Married 2. Single 3. Divorced 4. Widowed

B5. Your kids:

Options: 1. None 2. One 3. More than one (Please write down the total number of your kids)

B6. The land owned by your family including forests is ____ acres, and the land your family rented including forests is ____ acres.

Filled content: owned: 1.5 acres; rented: 1.5 acres

B7. In 2020, the total income of your family is ____, the agricultural income is ____, the costs for seeds, pesticides, fertilizers, irrigation are ____.

Filled content: total income: 70,000; agricultural income: 10,000; costs: 30,000

B8. Except the agricultural income, do you get extra income?

Options: 1. Yes 2. No (skip to C1)

If yes (multiple answers):Options: 1. Working in cities 2. Doing business 3. Subsidies from government 4. Salaries as governors/village officials/teachers 5. Aids from relatives and friends 6. Others (Please write down on the line)

**C Road Infrastructure Survey**

C1. Are all the roads from the village to the block hardened?

Options: 1. Yes 2. No

C2. Are all the roads from the village to the district hardened?

Options: 1. Yes 2. No

C3. When was the currently used road built?

Options: 1. Before economic reform (1991) 2. In the past 30 years 3. In the past 20 years 4. In the past 10 years 5. In recent 5 years

C4. The built village roads are mainly funded by ____.

Options: 1. Central government 2. Local (State/district/division) government 3. Loans from national or regional banks 4. Block panchayat/Village panchayat/Collective economy 5. Each household 6. Donation of villagers 7. Others (Please write down on the line) 8. I'm not sure

C5. If the village build a new road & funds will be collected from each household, how much are you expected to pay?

Options: 1. None at all 2. Below 1000 3. 1000-3000 4. 3000-5000 5. 5000~10000 6. Above 10000

C6. The current maintenance of village roads is done by ____.

Options: 1. District/Division department of transportation 2. Block panchayat 3. Village panchayat 4. I know someone maintain the village roads, but I can't say the specific department 5. I'm not sure if anyone maintains the village roads 6. No maintenance

C7. After the construction of roads to the block and district, how it can be mainly convenient to you? (Allow multiple answers)

Options: 1. It is more convenient to transport the agricultural products 2. It is more convenient to go to cities for work 3. It is conducive for the rural economy especially breeding and expanding characteristic economy such as rural tourism 4. It is more convenient for kids to go to school 5. Others (Please write down on the line)

C8. Compared to ten years ago, how do you think of the improvement in road construction in your village?

Options: 1. Better 2. Some progress 3. No change 4. Worse

C9. Compared to other block or district villages that you know, how do you think of the road construction in your village?

Options: 1. The best 2. Better 3. The same 4. Worse 5. The worst

C10. Are you satisfied with your village road?

Options: 1. Very satisfied 2. Relatively satisfied 3. Just so so 4. Relatively dissatisfied 5. Extremely dissatisfied

**D Drinking Water and Irrigation Infrastructure Survey**

D1. Does your family have access to tap water?

Options: 1. Yes 2. No (Skip to D8)

D2. When did your family connect to tap water?

Options: 1. Before economic reform (1991) 2. In the past 30 years 3. In the past 20 years 4. In the past 10 years 5. In recent 5 years

D3. How is your tap water priced?

Options: 1. Priced by cubic metres (/m³) 2. Priced by annual management fee (/year) 3. For free 4. Others (Please write down on the line)

D4. How do you think of the cost of the village tap water?

Options: 1. Very high 2. Relatively high 3. Suitable 4. Relatively low 5. Very low

D5. How do you think of the quality of the village tap water?

Options: 1. Very good 2. Good 3. Just so so 4. Relatively bad 5. Very bad

D6. How often does the tap water supply stop?

Options: 1. Never stop water supply 2. Occasionally stop water supply 3. Frequently stop water supply 4. Fixed time of water supply (How is the tap water supplied, please write down on the line)

D7. Who mainly provided the funds of existing tap water pipes and facilities in the village?

Options: 1. Central government 2. Local (State/district/division) government 3. Loans from national or regional banks 4. Block panchayat/Village panchayat/Collective economy 5. Each household 6. Donation of villagers 7. Others (Please write down on the line) 8. I'm not sure

D8. If no tap water, what's the source of your family drinking water?

Options: 1. Pressed well 2. Public well 3. Others (Please write down on the line)Filled content: Mole

D9. How many cubic meters of water does your family use for daily life per month?

Filled content: 25 m³/month

D10. If the village will build or rebuild the tap water facilities and the funds will be provided by each household, how much money you are expected to pay?

Options: 1. None at all 2. Below 1000 3. 1000~3000 4. 3000~5000 5. 5000~10000 6. Above 10000

D11. Compared to ten years ago, how do you think of the improvement of drinking water facilities in the village?

Options: 1. Better 2. Some progress 3. No change 4. Worse

D12. Compared to the other block or district villages, how do you think of drinking water facilities in your village?

Options: 1. The best 2. Better 3. The same 4. Worse 5. The worst

D13. Are you satisfied with the water for daily life in your village?

Options: 1. Very satisfied 2. Relatively satisfied 3. Just so so 4. Relatively dissatisfied 5. Very dissatisfied

D14. In 2020, the fee you pay for agricultural irrigation is totally ____, how do you think of its costs?

Filled content: 3000Options: 1. Very high 2. Relatively high 3. Suitable 4. Relatively low 5. Low

D15. In recent 10 years, how often has your farmland suffered droughts?

Options: 1. Never 2. Several times 3. Every year

D16. Which irrigation method does your family mainly adopt?

Options: 1. Water channel/canal/ditch 2. Sprinkling irrigation 3. Capillary irrigation 4. Drop irrigation/micro irrigation 5. Water irrigation by manpower 6. Natural rainfalls (Skip to D18)

D17. How do you pay for your annual irrigation fees?

Options: 1. An official of village panchayat is to collect door-to-door 2. The person entrusted and paid by village panchayat is to collect door-to-door 3. The contractor of the irrigation facilities is to collect door-to-door 4. Pay for the fees voluntarily 5. It depends

D18. The main water source of your irrigation is ____.

Options: 1. Reservoir (artificial lake) 2. Pump station 3. Motor-pumped well 4. Ponds 5. Others (Please write down on the line)

D19. When was the irrigation source you mainly use built?

Options: 1. Before 1947 2. Before 1991 3. In the past 30 years 4. In the past 20 years 5. In the past 10 years 6. In recent 5 years

D20. If the village will build or rebuild the irrigation facilities and the funds will be collected through each household, how much money you are expected to pay?

Options: 1. None at all 2. Below 1000 3. 1000-3000 4. 3000-5000 5. 5000~10000 6. Above 10000

D21. Compared to 10 years ago, how do you think of the improvement of the irrigation infrastructure in your village?

Options: 1. Better 2. Some progress 3. No change 4. Worse

D22. Compared to the other block or district villages, how do you think of the irrigation infrastructure in your village?

Options: 1. The best 2. Better 3. The same 4. Worse 5. The worst

D23. Are you satisfied with the irrigation infrastructure in your village?

Options: 1. Very satisfied 2. Relatively satisfied 3. Just so so 4. Relatively dissatisfied 5. Very dissatisfied

**E Other Village Public Goods and Service Survey**

E1. Have your family finished toilet revolution?

Options: 1. Yes (Please write down the finished year on the line, for example: 2001) 2. NoFilled content: 2009

E2. Is your family toilet water-washable?

Options: 1. Yes 2. No

E3. The main fuel used in your family for cooking is ____.

Options: 1. Natural gas 2. Electricity 3. Coal 4. Gas storage reservoir 5. Wood/straws/plant 6. Others (Please write down on the line)

E4. Has your family installed solar equipment?

Options: 1. Yes (Please write down the installation year on the line, Eg: 2001) 2. No

E5. Has your family installed cable television?

Options: 1. Yes (Please write down the installation year on the line, Eg: 2001) 2. NoFilled content: 2012

E6. Has your family accessed to broadband Internet service?

Options: 1. Yes (Please write down the accessed year, Eg: 2001) 2. No

E7. How is the mobile phone signal at your home?

Options: 1. Good 2. Bad 3. Sometimes good sometimes bad 4. No signal at all

E8. How mostly does your family go to block or district?

Options: 1. By foot 2. By Motorcycle 3. By bus or Tutu 4. By car

E9. Have you ever heard of any agricultural scientific & technological training program initiated by central or local governments?

Options: 1. Yes 2. No (Skip to E11)

E10. Have you ever participated in any agricultural scientific & technological training program initiated by central or local governments?

Options: 1. Yes (Please write down the times you participated on the line) 2. No

E11. Has your dwelling house been renovated in recent years?

Options: 1. Yes (Please write down the year on the line, Eg: 2001) 2. No (Skip to E13)Filled content: 2022

E12. Has the government granted a subsidy for the renovation of your dwelling house?

Options: 1. Yes 2. No

E13. Are you satisfied with the sanitation and medical care in rural areas?

Options: 1. Very satisfied 2. Relatively satisfied 3. Just so so 4. Relatively dissatisfied 5. Very dissatisfied

E14. Are you satisfied with the educational input in rural areas?

Options: 1. Very satisfied 2. Relatively satisfied 3. Just so so 4. Relatively dissatisfied 5. Very dissatisfied

E15. Are you satisfied with the social security input in rural areas?

Options: 1. Very satisfied 2. Relatively satisfied 3. Just so so 4. Relatively dissatisfied 5. Very dissatisfied

E16. Has the village panchayat initiated special control or management during the Covid-19 epidemic?

Options: 1. Yes 2. No

E17. Has the village panchayat ever publicized any epidemic prevention handbook or announcement during the Covid-19 epidemic?

Options: 1. Yes 2. No

E18. Has the village panchayat ever provided medical supplies like masks, hand-sanitizer during the Covid-19 epidemic?

Options: 1. Yes 2. No

E19. Are you satisfied with the prevention work in your village during the Covid-19 epidemic?

Options: 1. Very satisfied 2. Relatively satisfied 3. Just so so 4. Relatively dissatisfied 5. Very dissatisfied

E20. Have you always actively voted in the village panchayat election?

Options: 1. Yes 2. No

E21. Generally, are you satisfied with the management of your village panchayat?

Options: 1. Very satisfied 2. Relatively satisfied 3. Just so so 4. Relatively dissatisfied 5. Very dissatisfied

**F The Villagers' Needs of Public Goods Survey**

F1. Have you ever gave advice or opinions about village road construction, drinking water and irrigation infrastructure, etc. to any government department in charge?

Options: 1. Yes 2. No (Skip to F4)

F2. If yes, which way did you adopt? (Allow multiple answers)

Options: 1. Initiate or participate an agenda 2. Report to your village assembly (Gram Sabha) 3. Say to the villagers' representatives 4. Render a report to a local councilor or a member of parliament (MP) 5. Report to your village panchayat 6. Meet with the higher administrative authorities 7. Parade and demonstration 8. Sit-in protest 9. Others (Please write down on the line)

F3. If you have ever adopted any abovementioned action, have your action had impacts on the construction of the road or drinking water and irrigation infrastructure, etc.?

Options: 1. Yes 2. No 3. I'm not sure

F4. If you have some advice or opinions about village public goods supply, which way you'd like to adopt? (Allow multiple answers)

Options: 1. Initiate or participate an agenda 2. Report to your village assembly (Gram Sabha) 3. Say to the villagers' representatives 4. Render a report to a local councilor or a member of parliament (MP) 5. Report to your village panchayat 6. Meet with the higher administrative authorities 7. Parade and demonstration 8. Sit-in protest 9. Others (Please write down on the line)

F5. When drawing up a construction plan for a village road, have the government ever held a hearing or publicly consult with the villagers?

Options: 1. Yes 2. No 3. I'm not sure

F6. When drawing up a construction plan for village tap water and irrigation facilities, have the government ever held a hearing or publicly consult with the villagers?

Options: 1. Yes 2. No 3. I'm not sure

F7. When drawing up a construction plan for village infrastructures, do you agree that the government should hold a hearing or publicly consult with the villagers?

Options: 1. Yes 2. No 3. I'm not sure

F8. Due to the village road construction, who do you think should mainly provide the funds?

Options: 1. The central government 2. The state government 3. The district/division government 4. The block panchayat 5. The village panchayat 6. Each household 7. I'm not sure

F9. Due to the village drinking water and irrigation facilities construction, who do you think should mainly provide the funds?

Options: 1. The central government 2. The state government 3. The district/division government 4. The block panchayat 5. The village panchayat 6. Each household 7. I'm not sure

F10. Are the basic rural infrastructures such as road, water and irrigation satisfied with your needs of public goods?

Options: 1. Very satisfied 2. Relatively satisfied 3. Just so so 4. Relatively dissatisfied 5. Very dissatisfied

F11. Have you ever joined in any village organization such as self-help group, farmer specialized cooperatives?

Options: 1. Yes 2. No (Skip to F13)

F12. Are the village organizations you have joined in satisfied with your needs of education, healthcare, social security etc.?

Options: 1. Very satisfied 2. Relatively satisfied 3. Just so so 4. Relatively dissatisfied 5. Very dissatisfied

F13. If the construction funds of the village road need to be collected through every household, whose opinion you will adopt? (Allow three answers)

Options: 1. The clan elder 2. Relatives 3. Neighbors 4. President or leader of panchayat 5. Representatives of villagers 6. Government officials

F14. Which public goods do you think the village needs most? (Allow three answers)

Options: 1. Drinking water and irrigation facilities 2. Village road 3. Electricity supply 4. Toilet revolution 5. Renovation of dangerous old dwelling houses 6. Education 7. Health and medical care 8. Anti-poverty 9. Social security 10. Rural garbage treatment 11. Agricultural machinery 12. Convenient transportation
